# The role of leadership in empowering women in medicine

**DOI:** 10.3389/fpubh.2025.1607912

**Published:** 2025-07-23

**Authors:** Aline Yacoubian, Nada Khaddage-Soboh, Jad Najdi, Albert El Hajj

**Affiliations:** ^1^American University of Beirut Medical Center, Beirut, Lebanon; ^2^Suliman S. Olayan School of Business, American University of Beirut, Beirut, Lebanon

**Keywords:** women, leadership, medicine, female physician workforce, healthcare

## Abstract

Women continue to be understated and underestimated in leadership roles in science and medicine, where they face persistent gender bias and numerous challenges. This review paper seeks to detect the key barriers restricting female physicians’ access to leadership roles and to communicate key findings and recommendations from recent literature on gender equity in these disciplines. The review emphasizes substantial social barriers, including lack of support, mentorship, and sponsorship, alongside practical challenges such as excessive workload, childcare responsibilities, and limited career guidance, all of which deter leadership opportunities. The paper highlights the role of leadership in improving the visibility of female physicians and linking them to leadership opportunities. It also underlines the need for institutional funding through flexible work procedures, mentorship plans, and leadership enhancement plans, with the active participation of male leaders. The projected strategies aim to advance female physicians’ careers, challenge stereotypes about their leadership capabilities, and offer practical resources to enhance their representation in science and medicine—eventually fostering meaningful societal change.

## Background

The healthcare sector is “delivered by women and led by men” as described by the World Health Organization (WHO) for the distribution of responsibility between them in this domain (1). Gender equality typically means ensuring that everyone has the same rights, responsibilities, and opportunities (2). It includes reflecting the interests, needs, and priorities of both genders while acknowledging the diversity within women and men (2). The importance of assessing the status of gender inequality lies in its impact on medicine as it is so deeply rooted that it spills into biomedical research (3). Women form 70% of the global health and social workforce (4). Despite this, a significant gender gap remains evident in the senior leadership roles that women assume (5). Female underrepresentation in leadership positions persists despite some progress in the field. The goal remains to shatter the “glass ceiling”—the underrepresentation of women in leadership roles persists even in the absence of overt barriers (6) rather than merely raise it (1).

In other cases where women admitted to medical schools comprise around 50% of entering medical students, the disparity against female physicians is evident along with the need for professional equity (7). While leadership positions still do not reflect these changes, women made up of 50% of those specialized in pediatrics or pediatric subspecialties for the past 30 years and in 2018, 37% of full professors and 27% of pediatric department chairs were women (8). Accordingly, in the field of neonatology, women entering the field have been considered the majority for over 20 years with 75% women who are in the beginning year as neonatal-perinatal medicine fellows in 2018 (9). In contrast, the field of cancer palliative care presented more gender distribution with 50% of women entry level personnel and only 14% female physicians (10). In Canada, the first female dean in the medical school was appointed in 1999 which is 170 years after the medical school was initiated (11). This shows how the progression of women into leadership roles in academic medicine has lagged behind what would be expected based on their representation in the medical field over the past 35 years (6).

A report showed that male physicians repeatedly earn more than female physicians wherein women earn around 74 cents for each one-dollar men earn. This disparity results in remarkable career earnings gaps. Depending on the specialty, female physicians make between $0.9 million and $2.5 million less over the course of a 40-year career than male physicians. The projected career earnings difference for primary care physicians was $0.9 million, for non-surgical specialists it was $1.6 million, and for surgical specialists it was $2.5 million (12). Family doctors are also hypothesized to be mostly females, and much like nursing as women-dominated field, receive less support, remuneration, and attention since their voices are not represented, present, or heard within decision-making circles (13, 14).

## Methods

The narrative overview of current research on gender disparity and female leadership in healthcare and medicine served as the foundation for this work. A systematic search across databases such as PubMed and Google Scholar was performed to discover relevant research, encompassing works released from 2016 to 2025. Keyword combinations such “gender equity,” “female physicians,” “women in leadership,” “healthcare leadership,” and “barriers to advancement in medicine” were among the search terms.

Peer-reviewed papers, reviews, and reports about gender inequality in medical education or healthcare leadership that were published in English studies were required to meet the inclusion requirements. Literature that provides solid analysis or actual evidence regarding the institutional, societal, or structural difficulties that women in medicine face were considered. Publications written in languages other than English, opinion pieces devoid of empirical backing, and articles unrelated to healthcare leadership were among the exclusion criteria.

There were 48 publications chosen for full-text review after titles and abstracts were screened. Key obstacles and suggested remedies were identified through thematic analysis; recurrent themes included underrepresentation in leadership positions, lack of mentorship, workplace bias, unequal compensation, and the impact of social norms. In order to make practical suggestions, human resources tactics, legislative initiatives, and leadership development initiatives were also examined. The most pertinent manuscripts were chosen for this mini-review.

## Women in leadership positions

The general definition of leadership in the medical field is constantly evolving to ensure optimum safety and high-quality care by leading changes at all levels of the health system and achieving organizational goals (15). In general, women have made up 20 to 29% proportion of senior leadership positions between the years 2011 and 2020 but there has been slight improvement wherein nine out of 10 businesses worldwide now have at least one woman in their leadership teams (16). There remains a deficiency of gender parity at lower levels of leadership which blocks women’s progress through the leadership channel toward gender parity at senior leadership positions (1).

Leadership, however, in the medical field does not merely imply the Chief Executive Officer (CEO) status or the ability to make final calls but rather drive a team at each level through both clinical and non-clinical positions like team leader or board member (17). To do so, gender parity needs to be present at each level. Accordingly, a failure of women in leadership positions is evident as there remains a leaky pipeline and barriers at each level rather than in more senior positions (1).

While it is evident that women have less access to leadership positions in medical schools, editorial boards and authorships of academic journals and societies remain rare (10, 18). A study utilized the Composite Editorial Board Diversity Score (CEBDS) and reported that 57.5% were men from 1,219 editors (*p* < 0.001) and that 16 were women among 46 editors in chief identified (*p* < 0.05) (19). The Clarivate Analytics’ 2021 Journal Citation Reports showed that women were 1,177 from 4,898 members on editorial boards of highest oncology journals and 14 were from 71 editors-in-chief of the topmost oncology journals (20). As for the Middle East, a paper from Iraq indicated that around 20.18% of editorial board members were women and 4% of editor-in-chief were women with significant differences in editorial role according to gender (*p* < 0.0001) (21). These associations have related the significant female underrepresentation to unconscious biases and societal norms (18).

## What gender inequality looks like today: challenges encountered

The first study describing the standard image of a scientist was done by Mead and Metraux (22) among a population of American high-school students depicting the image of a scientist as a man who was middle aged or older with a beard and a white coat. This has been marked by significant improvement wherein in 2018 a meta-analysis of Draw-A-Scientist studies for five decades in the United States (US) reported that the likelihood of children depicting male scientists increased with age but declined over historical time in the US even though boys were more liable than girls to sketch male scientists (23). In Chambers’ breakthrough study (led between 1966 and 1977), less than 1% of children depicted a female scientist (Chambers). Later, studies conducted between 1985 and 2016 showed that the percentage increased to an average of 28% (23). Other papers show that male physicians were invited to give twice as many lectures about their research versus female physicians in a study examining colloquium speakers at 50 reputable universities (24). This implies that the underrepresentation of female experts sustains the concept that medicine or science are predominantly shaped and accessed by those who belong to the stereotype of a white male scientist (25).

More female physicians have been entering the workforce but continue to face constant challenges such as pay scale injustices, discrimination, and an inequality between obligations at work and home (26). Most women in healthcare are concentrated in roles with limited authority or influence over systems, organizations, or their professional advancement. This lack of autonomy often contributes to increased work-related stress, lower job satisfaction, and burnout—factors that can ultimately compromise the quality of patient care (26). Studies indicate the following challenges faced by women: Societal expectations wherein traditional cultural and gender roles, organizational culture that applies patriarchal and absence of mentorship and support (27). As for barriers encountered in Lebanon, these include family responsibility, economic crisis in Lebanon, and burnout as well as work-life imbalance (28). Microaggressions are a common way that gender bias in surgery still exists. The highest frequency and intensity of bias encounters and microaggressions were reported by trainees (29).

## Impact of absence of women in the field: importance of gender equality in the field

Studies have shown that gender inequality in the medical field has been translated into multiple dimensions within it. It is evident in the pay gap, implications for services, well-being, and value (27, 28). Most importantly, gender inequality becomes translated into health risks through prejudiced values, norms, principles, and practices; transcending into differential exposures and vulnerabilities to disabilities, injuries, and diseases; and further into health systems and health research biases. The multitude of impacts listed above portrays the detrimental impacts of gender discrimination on health and social outcomes (1). Following over 40 years of international dialogs on gender progress and more than a century of feminist advocacy, gender equality remains recognized as one of the most significant elements of health and economic advancement.

Gender biases in medicine undercut comprehensive economic progress, full employment, modest work and accomplishment of gender equality. These comprise ineffectiveness in health systems by restricting the efficiency, allocation, incentive and preservation of female employees, who form much of the health workforce. Further impacting medical staff, gender discrimination is related to reduced staff morale, decreased self-esteem, and lowered productivity. Gender inequality has become systematically incorporated in medical staff systems instigating further workforce maldistribution and ineffectiveness creating barriers to healthcare for those who may be most affected by it (30). This issue of equality and equity within the medical workforce is one that it is essential to tackle since it not only impacts its staff but also patient care and team performance (10). The medical field is no different, with prominent strong ethical disputes defending the need for gender equality in medicine since research indicates that gender equality in the clinical workforce can positively impact patient results (31).

## Examples from different countries

The status quo of women in the medical field and its consequences have had far-reaching implications over many years. This is tangibly evident in many cases around the world. The US has witnessed an increased percentage of female physicians with women making up 35% of the active physicians and 50% of US medical school graduates (32). The Organization for Economic Co-operation and Development (OECD) Health has presented statistics in 2021 that reflect that the ratio of female physicians in all OECD countries has increased during the last two decades from 45% in 2010 to 50% in 2020. This increase was specifically evident in the Netherlands, Spain, Denmark and Norway starting 2000. However, each country exhibits varying levels of female participation as Eastern European countries have higher percentages such as Latvia at 73.9% and Estonia at 73.2% (33).

On the other hand, Asian countries have extremely low representation of women at 24.5% in Korea and 22.7% in Japan. In the Swiss Society of Emergency and Rescue Medicine (SSERM), there was an increase in the proportion of women from 26 to 35% in the period of 2011 to 2021. The SSERM presents an exceptional example as the proportion of female physicians improved by 153% and women certified in prehospital emergency medicine by 131%. Within the analyzed emergency departments, women made up 50% of senior consultants and mentors with extended accountability by 2021. Regarding academics, the course of emergency medicine was reported by 11 Swiss universities to be taught by six full professors, of whom one was a woman in 2021 (34).

## The case in the Middle East

Gender discrimination in the Middle East is manipulated by cultural, religious, and socio-political factors, along with the lack of family-friendly guidelines that favor working mothers in maintaining a basic work-life balance. Moreover, ineffective organizational policies fail to eliminate or protect women from workplace discrimination. A comprehensive review of four electronic databases, including nine articles on gender equality in the Middle East about education and community pharmacy work practice, revealed that while educational opportunities were largely equal, this did not inevitably translate into equal job opportunities. Female representation in top pharmacy positions remains low. Additionally, these women earned less and were mandated to work in unpaid healthcare work (35). Some medical systems in the medical field portray a lack of support from leadership.

While it is universally portrayed that Middle Eastern women are understated in medical field leadership, there has been a rise in the human development index and women education completion levels. This has slowly become prevalent in all medical fields and within management levels (36). Despite worldwide initiatives to advance the Sustainable Development Goals (SDGs), ongoing conflicts and instability in the region pose remarkable barriers, especially in attaining health (SDG 3) and gender equality (SDG 5) (25). A study examined more than 1.7 million articles and indicated that men in the Middle East and North Africa (MENA) region higher research productivity and seniority even there are more female researchers in countries such as Algeria, Egypt, Lebanon, and Tunisia and that men publish on average between 11 and 51% more than women (37).

## The role of the leadership in cultivating gender equity

To break down structural obstacles and involve women in research and medicine with equal chances, leadership must be proactive change agents. This entails moving beyond identifying obstacles to offering a framework of quantifiable organizational-level solutions and emphasizing that achieving gender equity in leadership necessitates structural change (38).

Cooperation between institutions, leaders, and legislators is necessary to generate an inclusive and supporting atmosphere where women may flourish. Leadership should prepare and organize gender bias training to advance inclusive leadership and increase understanding of unconscious bias. This will eventually foster an inclusive culture through safe areas where women can express their concerns without worrying about comebacks. To gauge advancements, leadership ought to investigate and analyze female representation in leadership positions, hiring, and promotions on a consistent basis.

These are practical recommendations for medical centers to consider for promoting gender equity in medicine based on several studies (6, 19, 25, 39). The aim of this approach is to promote institutional surroundings that fully influence the talents of all faculty members and support a healthcare system that successfully meets the needs of patients.

1 Establish institutional assignments and responsibilities:

- Generate policies to ensure gender equity in pay, resource provision, promotion openings, and leadership positions.
- Introduce formal and informal mentoring and sponsorship programs to support female physicians in their professional careers and to avoid imposter syndrome.
- Evaluate and resolve inequalities in the workplace, create and implement gender equity audits.
- Offer programs for female physicians to receive proper leadership training.
- Encourage a positive work environment that stabilizes work-life balance for all staff members, decreasing the unequal workload for women.
- Determine leadership roles that facilitate practice efficiency, enhance working conditions, and maintain work-life balance.

2 Promote empowerment through education:

- Education plays a crucial role in integrating culturally responsive and diverse-conscious practices into the healthcare system.
- Promote autonomy and confidence in women by teaching them about their rights and giving them leadership and negotiating skills.
- Raise awareness of institutional unconscious bias through awareness campaigns to foster a more embracing work environment.
- Normalize pregnancy, maternity leave, back-to-work, and motherhood through offer offering flexible schedules, temporary part-time options when needed, and expanded childcare services if feasible.

3 Cultivate a positive cultural shift:

- Establish a platform for female physicians to improve their representation, tackle intersectionality in their professions, and enhance their professional growth in leadership. These initiatives revolve around offering mentorship, sponsorship, skill development, and career support.
- Eliminate long-standing gender preconceptions and gender bias with the need of a paradigm shift in career development and society cultures.
- Encourage gender-sensitive policies and candid conversations about equity to foster camaraderie.

4 The role of social media to empower women:

• Social media platforms can increase visibility of female physicians through showcasing the personal journeys, obstacles, and success strategies of female role models in medicine and illustrating diverse pathways to leadership.

• Highlight real-life experiences of encountering and winning systemic barriers for other amateur female physicians to receive valuable insights and encouragement into effective career progression.

• Encourage male leaders to act as advocates and supporters of gender equity in medicine and science.

• Build a robust network of both male and female physicians to promote gender equality and equity and foster diversity through sponsorship, mentoring, coaching, skill-building opportunities, and career guidance.

## Conclusion

Despite a constant increase in the number of female physicians over the past 30 years, attempts to address the challenges they face have not kept pace. Recognizing and defeating these barriers is a shared concern for female physicians internationally, remarkably in the Middle East. Advancing gender equity in medicine demands a more inclusive tactic, with leadership accenting that true equity imposes the active involvement of all individuals. Gender equity is not about prioritizing women over men but promoting collaboration between both genders to develop team dynamics and, eventually, enhance patient care and contribute to medical advancement.
